# Inhibitory Effects of Artificial Sweeteners on Bacterial Quorum Sensing

**DOI:** 10.3390/ijms22189863

**Published:** 2021-09-13

**Authors:** Victor Markus, Orr Share, Marilou Shagan, Barak Halpern, Tal Bar, Esti Kramarsky-Winter, Kerem Teralı, Nazmi Özer, Robert S. Marks, Ariel Kushmaro, Karina Golberg

**Affiliations:** 1 Department of Medical Biochemistry, Faculty of Medicine, Near East University, Nicosia 99138, Cyprus; victor.markus@neu.edu.tr; 2 Avram and Stella Goldstein-Goren Department of Biotechnology Engineering, Ben-Gurion University of the Negev, Be’er Sheva 84105, Israel; orrs@post.bgu.ac.il (O.S.); marilous@bgu.ac.il (M.S.); halpbar@post.bgu.ac.il (B.H.); bta@post.bgu.ac.il (T.B.); esti.winter@gmail.com (E.K.-W.); rsmarks@bgu.ac.il (R.S.M.); 3 Department of Medical Biochemistry, Faculty of Medicine, Girne American University, Kyrenia 99428, Cyprus; keremterali@gau.edu.tr; 4 Department of Biochemistry, Faculty of Pharmacy, Girne American University, Kyrenia 99428, Cyprus; nazmiozer@gau.edu.tr; 5 The Ilse Katz Center for Nanoscale Science and Technology, Ben-Gurion University of the Negev, Be’er Sheva 84105, Israel; 6 School of Sustainability and Climate Change, Ben-Gurion University of the Negev, Be’er Sheva 84105, Israel

**Keywords:** quorum sensing, artificial sweeteners, gut microbiota, normobiosis, dysbiosis

## Abstract

Despite having been tagged as safe and beneficial, recent evidence remains inconclusive regarding the status of artificial sweeteners and their putative effects on gut microbiota. Gut microorganisms are essential for the normal metabolic functions of their host. These microorganisms communicate within their community and regulate group behaviors via a molecular system termed quorum sensing (QS). In the present study, we aimed to study the effects of artificial sweeteners on this bacterial communication system. Using biosensor assays, biophysical protein characterization methods, microscale thermophoresis, swarming motility assays, growth assays, as well as molecular docking, we show that aspartame, sucralose, and saccharin have significant inhibitory actions on the Gram-negative bacteria *N*-acyl homoserine lactone-based (AHL) communication system. Our studies indicate that these three artificial sweeteners are not bactericidal. Protein-ligand docking and interaction profiling, using LasR as a representative participating receptor for AHL, suggest that the artificial sweeteners bind to the ligand-binding pocket of the protein, possibly interfering with the proper housing of the native ligand and thus impeding protein folding. Our findings suggest that these artificial sweeteners may affect the balance of the gut microbial community via QS-inhibition. We, therefore, infer an effect of these artificial sweeteners on numerous molecular events that are at the core of intestinal microbial function, and by extension on the host metabolism.

## 1. Introduction

For many years, bacteria have been thought to act independently. The discovery that they act collectively through a sophisticated network of cell-cell communication, known as quorum sensing (QS) has been a great leap in the understanding of bacterial biology [1]. The QS system enables bacteria to interact and adjust their gene expression based on their population density. Most Gram-negative bacteria use the *N*-acyl homoserine lactone (AHL)-mediated QS system [2], while others, such as the opportunistic pathogen *Pseudomonas* *aeruginosa,* utilize alkyl quinolone (AQ) in addition to the AHL-mediated QS [3,4]. Gram-negative bacteria typically use AHL signals for QS, molecules which diffuse out of the cell and into the local environment [5,6]. Signal concentration increases with population density, and once the AHL signal concentration reaches a threshold level within the cells, these signals will bind their cognate intracellular receptors, the LuxR-type family of transcriptional regulators. The activated LuxR-type receptors, then alter gene expression levels to initiate behaviors that will benefit the group and/or are only achievable as a bacterial community. These include a range of virulence phenotypes such as biofilm formation, protease, and toxin production, and motility mechanisms [7]. In Gram-positive bacteria, QS is mediated using peptides as signal molecules [8,9]. Other QS systems identified in bacteria use the signaling molecules known as universal signal autoinducer-2 (AI-2) or autoinducer-3 (AI-3). The latter is produced by intestinal bacterial species including enterohemorrhagic *E. coli* (EHEC) to regulate its pathogenesis [10]. Emerging evidence from the human gut ecosystem reveals a new and prominent AHL signaling molecule (3-oxo-C12:2-HSL) associated with normobiosis and bowel health [11]. Patients with active inflammatory bowel disease (IBD), exhibit lower levels of 3-oxo-C12:2-HSL corresponding to an imbalance in the gut microbiota that is characterized by a significant reduction in Firmicutes (*C. coccoides*, *C. leptum*, and *F. prausnitzii*) and a significant rise in *Lactobacilli* count compared to the healthy individuals [11]. Follow-up studies confirm a link between 3-oxo-C12:2-HSL and normobiosis [12,13], and an anti-inflammatory effect on enterocytes without interfering with the paracellular permeability [12].

Artificial sweeteners, also known as non-nutritive sweeteners (NNS) and commonly used in many types of foods and beverages, are recommended for bodyweight reduction and persons living with type 2 diabetes mellitus and glucose intolerance [14]. Although the United States Food and Drug Administration (FDA) approved the use of aspartame, saccharin, sucralose, acesulfame potassium (Ace-K), advantame, and neotame, there is still no consensus in the scientific community regarding the safety status of these artificial sweeteners. Most studies using rodent models indicate deleterious effects of artificial sweeteners on metabolic health [15,16,17,18]. In humans, even though randomized controlled trials (RCT) suggest bodyweight benefits from the consumption of artificial sweeteners, there is still only limited data on other metabolic alterations [19], including altering gut microbiota in a manner that could enhance the metabolic diseases that they were intended to reduce [15,17,18].

The link between AHL and normobiosis [11,12,13,20,21] suggests that interference in the AHL-based communication network could influence gut microbiota balance and function. This study, therefore, aims to evaluate their impact on the AHL-based communication network. Due to the unavailability of commercially synthesized 3-oxo-C12:2-HSL, we used structurally similar AHL signaling molecules. 

## 2. Results

### 2.1. Effect of Artificial Sweeteners on Bioreporter Strains

**Figure 1:**
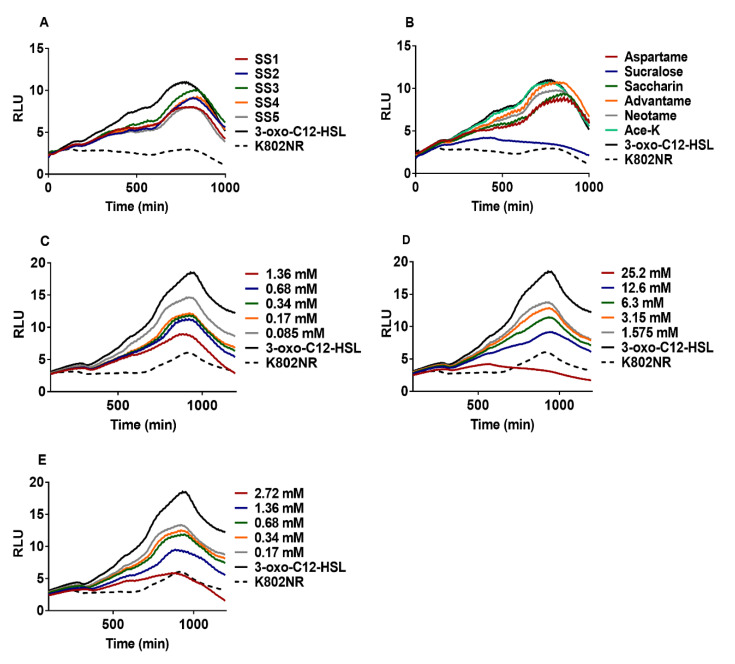
Anti-QS activity of artificial sweeteners. (**A**) Effect of different SS products on the K802NR bioreporter. SS1 (0.2 mg mL^–1^) contains sucralose, SS2 (0.4 mg mL^–1^) contains Ace-K and sucralose, SS3 (0.2 mg mL^–1^) contains Ace-K and sucralose, SS4 (0.3 mg mL^–1^) contains Ace-K and sucralose, and SS5 (0.3 mg mL^–1^) contains Ace-K and sucralose. The amounts of artificial sweeteners in the products are undisclosed. (**B**) Screening of six pure FDA-approved artificial sweeteners: aspartame (1.36 mM), sucralose (25.2 mM), saccharin (2.72 mM), advantame (0.42 mM), neotame (0.53 mM) and Ace-K (4.97 mM). (**C**) The response of K802NR to different concentrations of aspartame. (**D**) The response of K802NR to different concentrations of sucralose (**E**) The response of K802NR to different concentrations of saccharin. All concentrations of artificial sweeteners presented are final concentrations. The final concentration of 3-oxo-C12-HSL used was 5 × 10^–10^ M. The luminescence of K802NR was expressed as RLU.

To assess the potential of artificial sweeteners to disrupt the AHL-based communication circuit, we used *Escherichia coli* K802NR, a recombinant bioluminescent reporter strain harboring the plasmid pSB1075 infused with the QS promoter *lasI* that controls the transcription of the *V. fischeri luxCDABE* and *P. aeruginosa lasRI* genes. The presence of 3-oxo-C12-HSL induces the production of a light signal by K802NR. Residual low “background” light was produced by K802NR in the absence of 3-oxo-C12-HSL. Since athletes pay attention to their diet and use supplements to improve their performance in training sessions and competitions, we hypothesized that they may be the highest consumers of artificial sweeteners, because many of the supplements they use contain artificial sweeteners in an undisclosed amount. We evaluated the effect of sports supplement (SS) products on K802NR. The SS products contained at least one of the artificial sweeteners approved by the FDA (Table S1). The results obtained indicate that all the SS products significantly decreased the bioluminescence emission of K802NR relative to the control (Figure 1A and Figure S1A).

To ascertain the inhibitory activities of the artificial sweeteners in the SS products on K802NR, we used pure samples of six FDA-approved artificial sweeteners and tested them against K802NR. Of the six artificial sweeteners screened (aspartame, saccharin, sucralose, acesulfame potassium (Ace-K), advantame, and neotame), only three (aspartame, sucralose, and saccharin) were found to exert significant inhibitory activity against K802NR (Figure 1B and Figure S1B). Interestingly, all the SS products we analyzed contained one of these three artificial sweeteners. To understand whether these three artificial sweeteners affect bacterial growth, we measured the absorbance at 600 nm (OD_600_) of K802NR in the presence of varying concentrations of the artificial sweeteners with respect to time. The results revealed that the artificial sweeteners were not bactericidal (Figure S2). We then conducted a second experiment with PAO-JP2 (pKD-*rhlA*), a *lasI-rhlI* double mutant of *P. aeruginosa* PAO1 containing the plasmid pKD infused with C4-HSL responsive *rhlA* promoter coupled upstream to the *luxCDABE* box (Figure S3). Except for sucralose, the agents appeared ineffective in reducing bioluminescence emission of the bioreporter within the tested concentrations. Overall, these results suggest that aspartame, sucralose, and saccharin exert inhibitory activities prominently via LasR interference with sucralose affecting the *rhl* system.

### 2.2. Artificial Sweeteners Decrease LasR Solubility

**Figure 2:**
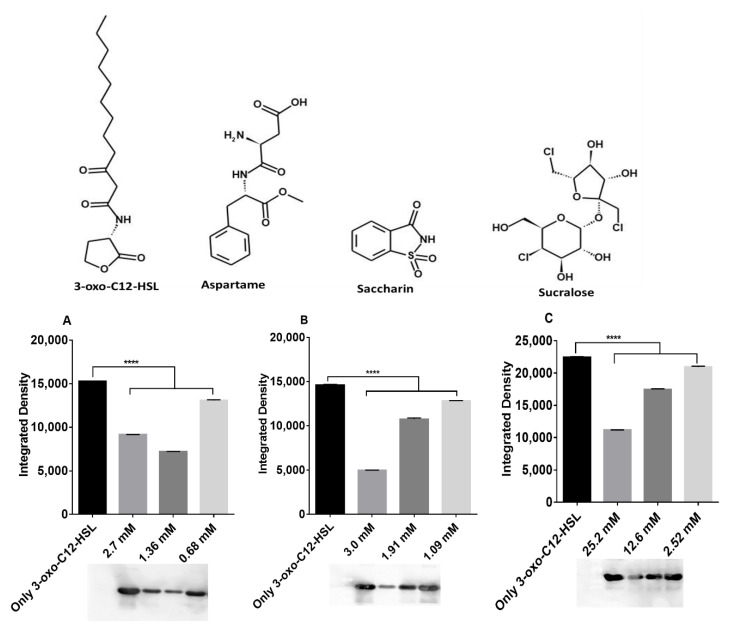
Decrease of LasR-LBD solubility by artificial sweeteners monitored by Western blotting of the soluble fraction from the LasR-LBD overexpression in *E. coli* BL21 strain in the presence of (**A**) aspartame, (**B**) saccharin, and (**C**) sucralose. All concentrations presented are final concentrations. The final concentration of 3-oxo-C12-HSL used was 100 nM. Integrated density is the sum of the values of the pixels within the protein band determined using ImageJ software [22]. **** *p* < 0.0001. Values represent mean ± SD, *n* = 3. Structures were drawn using MedChem Designer, Version 5.5 (Simulations Plus Inc., Lancaster, CA, USA).

The discovery that aspartame, sucralose, and saccharin exert their inhibitory effects prominently via LasR prompted us to seek to further clarify the molecular mechanism of their action. We used *E. coli* BL21, a strain transformed with the pETM-11 vector encoding a shortened His_6_-tagged LasR construct, LasR-LBD (ligand-binding domain) spanning residues Met1 to Lys173. We expressed the LasR-LBD protein in the presence of the 3-oxo-C12-HSL and/or the artificial sweeteners. After purifying the protein using nickel-affinity chromatography, LasR-LBD was monitored by SDS–PAGE and Western blotting. Without any ligand, except 0.15% (*v/v*) DMSO, no LasR protein was detected in the supernatant, but the addition of 7.5 μM 3-oxo-C12-HSL caused a significant expression of LasR (Figure 2). On the other hand, the artificial sweeteners were observed to significantly reduce the expression of LasR relative to the control, indicating that the solubility of the receptor was decreased (Figure 2).

### 2.3. Microscale Thermophoresis (MST)

**Figure 3:**
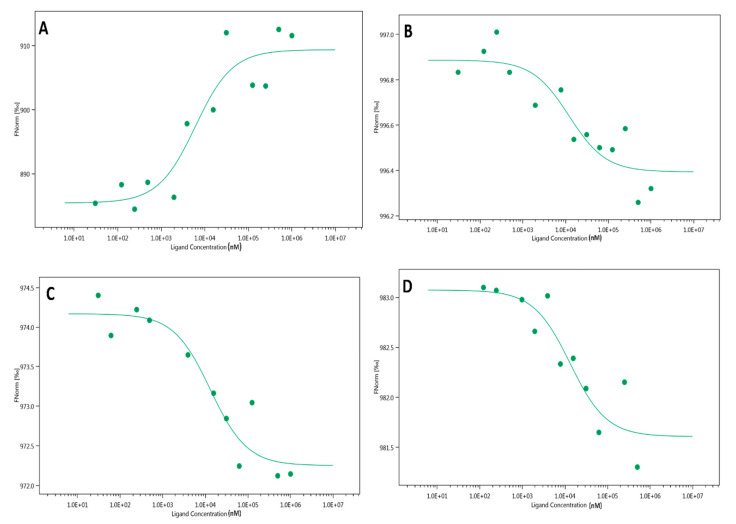
MST analysis of the interaction between artificial sweeteners and the LasR protein. (**A**) 3-oxo-C12-HSL binds LasR with a *K_d_* of 5823.3 ± 4.17 nM. (**B**) aspartame binds LasR with a *K_d_* of 11,845 ± 0.11 nM. (**C**) Sucralose binds LasR with a *K_d_* of 12,835 ± 0.29 nM (**D**) saccharin binds LasR with a *K_d_* of 12,896 ± 0.27 nM.

To perform MST, first, the LasR protein was expressed in recombinant *E. coli* and purified in the absence of AHL. The protein was then fluorescently labeled and titrated against aspartame, saccharin, sucralose, and 3-oxo-C12-HSL. The titration of the ligands induced fluctuations in the molecular properties of the fluorescently labelled protein observed in a temperature gradient, which was tracked using MST. The data obtained were used to plot binding curves, with the fitted *K_d_* of 3-oxo-C12-HSL lower than those of the artificial sweeteners, as shown in Figure 3, demonstrating its higher affinity to LasR.

### 2.4. Swarming Motility Assay

**Figure 4:**
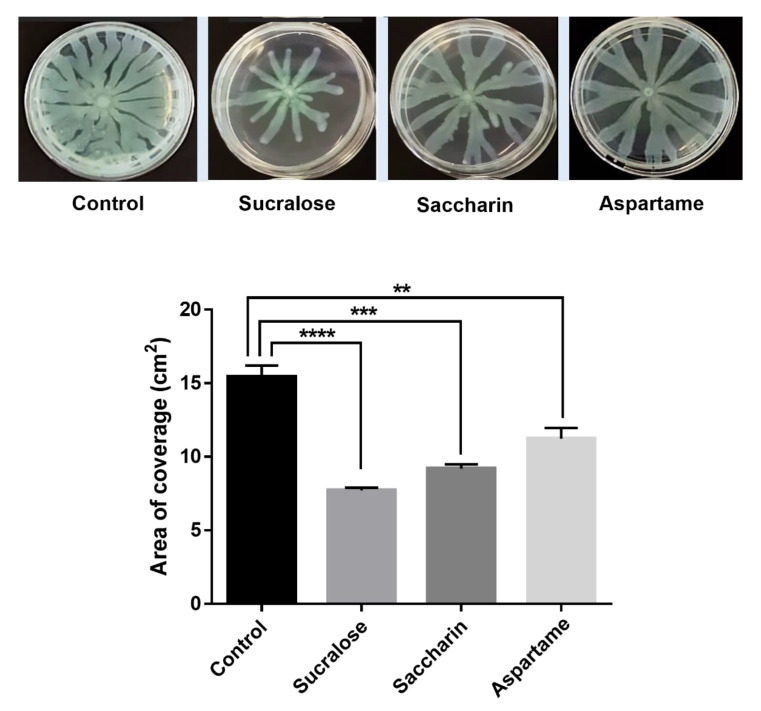
Alteration of bacterial translocation in *Pseudomonas aeruginosa* PAO1 by artificial sweeteners compared with the control. Sucralose (25.2 mM), saccharin (2.72 mM), and aspartame (1.36 mM) show significant inhibitory effects on bacterial motility. ** *p* < 0.01, *** *p* < 0.001, and **** *p* < 0.0001.

Since bacterial translocation along different surfaces is controlled hierarchically by the LasR machinery, we tested the inhibitory impact of the artificial sweeteners (aspartame, sucralose, and saccharine) by performing a swarming motility assay using *P. aeruginosa* PAO1. The motility of the bacteria across the soft agar shows that the three artificial sweeteners have significant inhibitory activities (Figure 4).

### 2.5. QS Competition Assay Using Chromobacterium Violaceum CV026

To further assess the inhibitory impact of aspartame, sucralose, and saccharine on AHL-based QS, we used *CV026.* The production of violacein, a purple pigment, by *CV026* in response to QS signaling molecule *N*-hexanoyl homoserine lactone (C6-HSL), was tested in the presence and absence of artificial sweeteners. Here, at higher concentrations than expected, only saccharin inhibited violacein production (Figure S4). 

### 2.6. In-Silico Studies

**Figure 5:**
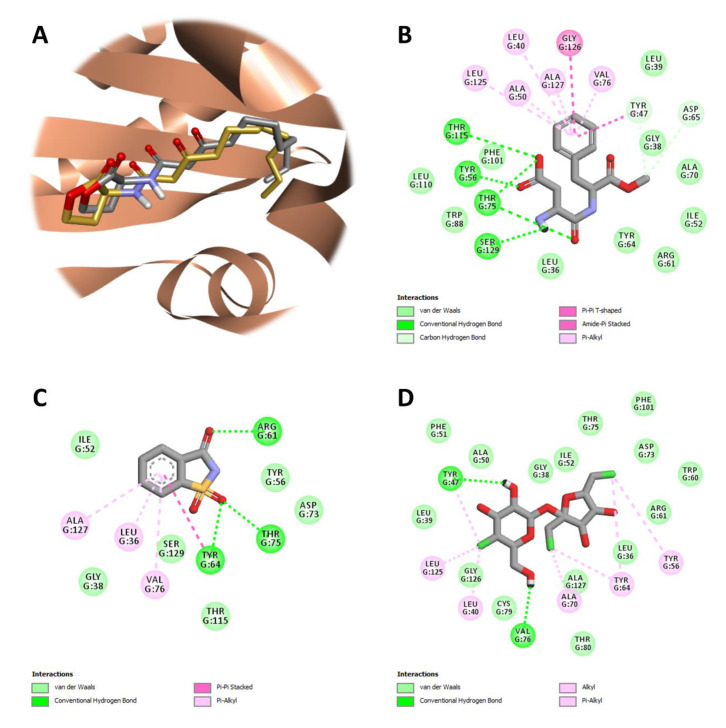
Protein-ligand docking and interaction profiling. (**A**) Close-up view of the superposed structures of co-crystallized (gold) and redocked (silver) 3-oxo-C12-HSL in the ligand-binding cavity of LasR (PDB ID: 2UV0; chain ID: G). Favorable non-covalent interactions stabilizing the relative position of (**B**) aspartame, (**C**) saccharin, or (**D**) sucralose with respect to the LasR-LBD. Images were prepared and rendered using Discovery Studio Visualizer, version 16.1.0 (Dassault Systèmes BIOVIA Corp., San Diego, CA, USA).

The docking protocol was validated with respect to reliability by redocking the co-crystallized ligand, 3-oxo-C12-HSL, into the ligand-binding site of the prepared structure of LasR. The co-crystal pose of the autoinducer was reproduced remarkably well, with a root-mean-square (r.m.s.) deviation of 0.587 Å over 21 matching heavy-atom pairs (Figure 5A).

The estimated binding affinity of the top-ranking docking solution was −9.0 kcal mol^–1^. 3-Oxo-C12-HSL has been reported to establish hydrogen-bonding interactions with Tyr56, Trp60, and Ser129 through its ketone groups, as well as with Asp73 and Thr75 through its amide group. Also, a water molecule appears to bridge Arg61 with the 3-oxo ketone group. The long acyl chain of the autoinducer is housed in a large hydrophobic pocket on the opposite side of the ligand-binding site [23]. In cross-docking calculations, three artificial sweeteners (aspartame, saccharin, and sucralose) that exhibited significant QS inhibition in test bacteria, were docked onto the prepared receptor (LasR) to gain a deeper understanding of the most favored binding site and the major receptor-ligand interactions responsible for the antagonism of the artificial sweeteners. Accordingly, aspartame was found to bind at the ligand-binding site of LasR, with a docking score of −8.6 kcal mol^–1^ (Figure 5B). Here, it was able to form hydrogen-bonding interactions with Tyr56, Thr75 and Thr115 through its carboxyl group, as well as with Ser129 through its amide group. The carboxyl group also appeared to accept a hydrogen bond from Thr75, the latter which could foster another hydrogen bond with one of the two ketone groups of aspartame. The phenyl ring of aspartame occupied the hydrophobic pocket of LasR, engaging in hydrophobic interactions with Leu40, Tyr47, Ala50, Val76, Leu125, Gly126 and Ala127. The methoxy group of aspartame was able to establish weak (non-conventional) hydrogen bonds with both Tyr47 and Asp65. As in the case of aspartame, the ligand-binding site was predicted by the docking algorithm to be the most favored cavity for saccharin binding (Figure 5C). The calculated binding affinity of the top-ranking docking solution was −7.3 kcal mol^–1^. Saccharin was able to form hydrogen-bonding interactions with Tyr64 and Thr75 through one of its two oxo substituents (at position 1), as well as, with Arg61 through its keto-group (at position 3). The benzene ring of saccharin was involved in hydrophobic interactions with Leu36, Tyr64, Val76 and Ala127 from the hydrophobic subsite of LasR. Like aspartame and saccharin, sucralose binding favored the ligand-binding site of the receptor, with a docking score of −6.1 kcal mol^–1^ (Figure 5D). The 4-chloro-4-deoxy-d-galactose (4-CG) unit of saccharin was able to engage in hydrogen-bonding interactions with Tyr47 and Val76 through its hydroxyl groups. The position, orientation, and conformation of this artificial sweetener relative to LasR were further stabilized by hydrophobic interactions occurring between the 4-CG unit and Leu40, Tyr47 and Leu125, as well as, between the 1,6-dichloro-1,6-dideoxy-d-fructose (1,6-DCF) unit and Tyr56, Tyr64, and Ala70.

## 3. Discussion

The occurrence of QS in the gut microbiota [13,24,25] suggests that it could play a role in normobiosis. By monitoring signal molecules, such as AI-2 and AHL, researchers revealed that bacterial interactions influence the composition of the gut microbiota and affect the balance of their community [13,24,26,27]. Although the effects of artificial sweeteners, such as sucralose, saccharin, and aspartame on bacterial populations have previously been demonstrated [28,29,30], the mechanism by which these sweeteners impact their inhibitory effects has not been fully understood. Therefore, we hypothesized that one possible cause for such alterations might be due to QS-inhibition. In the present study, we demonstrate that aspartame, sucralose, saccharin, and SS products exert anti-QS activity by disrupting the AHL-based communication network in our model bacteria. Using bioreporters carrying LasR or RhlR circuitry, we show that aspartame, sucralose, and saccharin exert their inhibitory activities significantly via LasR, with sucralose affecting RhlR. An important note is the fact that none of the sweeteners appear to have a bactericidal effect. 

Since the ability of bacteria to translocate along different surfaces is regulated hierarchically by the LasR system [5], we further tested the effect of aspartame, sucralose, and saccharin on *P. aeruginosa* PAO1 motility. Because aspartame, sucralose, and saccharin showed significant inhibitory effects on LasR, we speculated that similar effects might also be observed in bacterial translocation. Indeed, we show that these sweeteners inhibit the swarming motility of *P. aeruginosa* PAO1. The bacteria showed less branching and less expansion when incubated with each one of the sweeteners relative to control. These results emphasize the effect of artificial sweeteners on bacterial communication and in particular the effects on the different phenotypes regulated by the QS master receptor LasR. Moreover, the inhibition of violacein production in *CV026 by* saccharin and the disruption of *rhl* system by sucralose further underscore the effects these artificial sweeteners have on AHL-dependent QS circuitry. 

Using LasR as a representative receptor, we sought further to clarify the mechanism of action of the sweeteners. We demonstrated that aspartame, sucralose, and saccharin exert their inhibitory effects by interfering with the solubility of the protein. In the absence of the native ligand, LasR and related proteins are known to remain insoluble and do not fold; however, in the presence of the native ligand, LasR and related proteins fold and become soluble [31,32,33,34,35,36,37]. The binding of 3-oxo-C12-HSL to the LasR receptor activates the signal transduction cascade that results in the expression of the target genes [4]. Accordingly, the LasR protein was found to be expressed significantly in the presence of 3-oxo-C12-HSL. However, there was a significant decrease in the expression of the protein following the addition of artificial sweeteners. The artificial sweeteners appear to interfere with the binding of 3-oxo-C12-HSL to the LasR receptor. 

To assess the molecular interactions between the LasR and the artificial sweeteners, we used the MST technique which requires neither surface immobilization nor large sample sizes to perform. This has great advantages over conventional methods such as isothermal titration calorimetry (ITC) and surface plasmon resonance (SPR) [38]. As expected, the *K_d_* of 3-oxo-C12-HSL was comparatively lower than those of the artificial sweeteners, suggesting that 3-oxo-C12-HSL has a higher affinity to LasR than the artificial sweeteners. Different authors [39,40] have reported different *K_d_* values for 3-oxo-C12-HSL than the one obtained in this study, which could be explained by the disparity in the methodologies used. A notable difference in the normalized fluorescence of the artificial sweeteners was the downward trend, suggesting that the fluorescent molecules diffuse toward the heat focus. However, an upward trend of the normalized fluorescence was observed with 3-oxo-C12-HSL treatment. It has been suggested that the upward trend of normalized fluorescence, where the fluorescent molecule diffuses away from the heat focus, could be a result of the irreversible binding mode [40]. The ultra-tight binding of 3-oxo-C12-HSL to LasR has been earlier suggested [34]. 

Our in-silico studies suggest that aspartame, saccharin, and sucralose compete with the cognate ligand for binding at the ligand-binding site of LasR. They can all be accommodated well in this ~505-Å^3^ cavity of the molecule, thereby exerting an antagonistic effect on LasR-mediated cell-cell communication. One of the most challenging aspects of QS modulation is the distinction between an agonist and an antagonist. A flexible loop conventionally called L3 (residues 40 to 51) near the ligand-binding site, has been shown to be a deformable region enabling LasR to accommodate ligands with larger substituents (such as longer acyl chains), and the packing of Tyr47 against the autoinducer, which, in particular, has been shown to shield the ligand-binding site from bulk solvent [32,41]. Site-directed mutagenesis studies coupled with reporter gene activity assays have revealed that Tyr56, Trp60, and Ser129 represent critical residues in determining whether non-lactone QS modulators behave as agonists or antagonists of LasR [42]. Computational studies have demonstrated that the presence or absence of favorable interactions between AHL analogs and the hydrophobic subsite of LasR (which is lined by Tyr64, Val76, Trp88, Leu125 and Ala127) is also crucial to the activities of these ligands, as agonists or antagonists [43]. On the other hand, from a ligand-wise perspective, it has been stated that even minor variations in the structure of a potent QS modulator can have unique and significant effects (either towards agonism or towards antagonism) on signaling [44]. Aspartame, saccharin, and sucralose seem to interact simultaneously with at least a few of the critical and decisive residues (Tyr47, Tyr56, Arg61, Thr75 and Ser129) that are involved in stabilizing LasR–ligand complexes. However, they do not carry a lengthy hydrophobic substituent that can fill in the hydrophobic pocket of the receptor. This may introduce structural instabilities into the system, interfering with proper folding and rendering the receptor insoluble. In other words, these artificial sweeteners appear to have pharmacophore features, required by LasR for ligand reception, but they probably fail to yield a soluble complex with correct folding and full biological activity. We believe that structural investigations into receptor-ligand interactions are needed to better understand productive ligand-binding modes that activate or inhibit a receptor, as well as receptor conformational changes that occur upon ligand binding. These investigations may pave the way for the design of novel modulators that effectively target LuxR-type transcriptional regulators. Similarly, and related to this, more research is required to generate three-dimensional agonist- and antagonist-selective pharmacophores that cover most of the small-molecule (natural, semi-natural, or artificial) chemical space. These three-dimensional pharmacophores may eventually be used as a predictor of the modulatory action of LuxR-type regulator ligands on the QS circuitry.

Considering the role of AHL signaling in normobiosis [11,12] together with the findings reported in the present study, it is enticing to speculate that these artificial sweeteners could interfere with gut microbiota homeostasis, thus promoting the progression of digestive diseases. Our speculation is reinforced by the evidence indicating that patients with active IBD have significantly less 3-oxo-C12:2-HSL (16%) compared to healthy individuals (65.4%) [13]. Other studies indicate that IBD is associated with gut dysbiosis with a marked decrease in specific taxonomic groups including Firmicutes and Bacteroidetes. These gut residents are known to be involved in the regulation of the immune system, production of vitamins, facilitation of dietary substrates digestion, and repression of pathogens expansion [45,46]. These beneficial functions of the gut microbiota are likely to be hampered in the event of artificial sweetener-induced dysbiosis. The AHL molecule used in the present study (3-oxo-C12-HSL) has been shown to have anti-inflammatory effects on enterocytes [26], similar to its 3-oxo-C12:2-HSL counterpart [12], suggesting that the observations in this study might reflect what could occur in the gut ecosystem exposed to these artificial sweeteners. It has been suggested that the 3-oxo-C12:2-HSL molecule functions by not only protecting the enterocytes, but also directly enhancing the activities of Firmicutes [12,20,21]. Indeed, the Firmicute *Faecalibacterium prausnitzii* has been shown to have anti-inflammatory properties through the activation of NF-κB and by stimulating the synthesis of IL-8 [47]. In the absence of concrete scientific evidence to support or refute the safety of artificial sweeteners, more people, including children and adolescents, are consuming the agents in larger quantities [48]. Reports have shown that the number of products in the market containing artificial sweeteners has quadrupled over the years [48] as governments have increased the regulations to lower glucose intake of drinks and foods by manufacturers. Yet it has become difficult to estimate the concentration of artificial sweeteners in products because manufacturers may not specify the content, making it challenging to better assess the quantity of these artificial sweeteners consumed by individuals or population and the consequent health effects [48]. The present work underpins the potential of artificial sweeteners to disrupt gut microbiota homeostasis through their effects on quorum sensing. Therefore, there is a need to continue to elucidate the mechanisms of action of the effects of these sweeteners and other related products on gut microbiota [49,50]. Further studies that address the limitations of this work are recommended. Our focus in this study was on a few bacterial models, suggesting that the results obtained may not give the whole picture of the gut microbiota where trillions of different microbial species dwell together.

## 4. Materials and Methods

### 4.1. Bacterial Strains

In this study, the recombinant bioluminescent *Escherichia coli* K802NR strain (obtained from Davies, J. University of British Columbia, Canada) harboring the plasmid pSB1075 infused with the QS promoter *lasI* that controls the transcription of *Vibrio fischeri luxCDABE* and *Pseudomonas aeruginosa lasRI* genes, was used for detecting the inhibitory impacts of artificial sweeteners by monitoring the bioluminescent emission. A secondary experiment was performed with PAO-JP2 (pKD-*rhlA*), a *lasI-rhlI* double mutant of PAO1 containing the plasmid pKD infused with the C4-HSL responsive *rhlA* promoter coupled upstream to the *luxCDABE* box (obtained from Meijler M. M. Ben-Gurion University of the Negev, Be’er Sheva, Israel). The wild–type *Pseudomonas aeruginosa* PAO1 was used for the swarming motility assay [51]. Finally, *Chromobacterium violaceum* (CV026) strain was used for QS competitive assay [52]. The strain stocks were stored at −80 °C in 50% (*v/v*) of glycerol, a cryoprotectant additive.

### 4.2. Strain Cultivation

Stock K802NR strain was grown on LB-agar plates (Difco Luria-Bertani medium, BD) containing 100 μg mL^–1^ ampicillin for 48 h at 37 °C in an incubator (Binder, Camarillo, CA, USA). A single colony from the LB-agar plates was selected and introduced into a 10 mL LB medium containing 100 μg mL^–1^ ampicillin and grown overnight at 37 °C with shaking (140 rpm) on a rotary thermo-shaker (Gerhardt, Konigswinter, Germany) to provide aeration. Then, a 20 mL fresh portion of LB medium without antibiotic was inoculated with 20 μL of the inoculum from the overnight culture and re-grown at 30 °C for about 4 h until the early log phase (OD_600_ ≈ 0.2) without shaking. The OD_600_ was determined by Ultrospec 2100 pro spectrophotometer (Amersham, Berks, UK). As for the PAO-JP2 (pKD-*rhlA*) strain, it was introduced from the stock on LB-agar plates (Difco Luria-Bertani medium, BD) containing 300 μg mL^–1^ trimethoprim for 24 h at 37 °C in the incubator (Binder, Camarillo, CA, USA). From the LB-agar plates, a single colony was picked and introduced into 10 mL LB medium supplemented with 300 μg mL^–1^ trimethoprim and grown overnight at 37 °C with shaking (140 rpm) on a rotary thermo-shaker (Gerhardt, Konigswinter, Germany). The respective LB-agar plates containing K802NR and PAO-JP2 (pKD-*rhlA*) strains were stored at 4 °C for future use.

### 4.3. Bioluminescence Assay

Luminescence from the K802NR bioreporter strain was measured in a white opaque 96-well microtiter plate containing 80 μL of the bacterial culture (OD_600_ ≈ 0.2), 10 μL of 3-oxo-C12-HSL (at a final concentration of 5 × 10^–10^ M), and 10 μL of the different concentrations of artificial sweeteners or sports supplements. The positive control contained 10 μL 3-oxo-C12-HSL and 10 μL LB medium in addition to the bacterial culture, and the negative control contained 20 μL LB in addition to the bacterial culture. The luminescence was measured at 5 min intervals by Luminoskan Ascent Luminometer (Thermo Fisher Scientific, Waltham, MA, USA), maintained at 490 nm and temperature of 26 °C for 21 h with continuously shaking. As for the PAO-JP2 (pKD-*rhlA*) strains, the luminescence was measured in a white opaque 96-well microtiter plate containing 80 μL of the bacterial cultures (OD_600_ = 0.015), 10 μL of the different concentrations of artificial sweeteners, and 10 μL of C4-HSL (at a final concentration of 10 μM). The luminescence plate measurements were performed at 10 min intervals for 21 h at 490 nm and 37 °C with continuous shaking. Luminescence of the strains was expressed in relative light units (RLU). Concentration ranges of artificial sweeteners used in this study were calculated to reflect the concentrations within FDA-approved acceptable daily intake (ADI) for each of the chemicals.

### 4.4. Growth Assay

The effect of the artificial sweeteners on bacterial growth was measured in transparent 96-well flat-bottom microtiter plates (Corning) containing 80 μL of the bacterial culture (OD_600_ ≈ 0.2), 10 μL of 3-oxo-C12-HSL (at a final concentration of 5 × 10^–10^ M), and 10 μL of the different concentration of artificial sweeteners using an Ultrospec 2100 pro spectrophotometer (Amersham, Berks, UK). The positive control contained 10 μL 3-oxo-C12-HSL and 10 μL LB medium in addition to the bacterial culture, and the negative control contained 20 μL LB in addition to the bacterial culture. The OD_600_ was measured with continuous shaking of the plates maintained at 26 °C for 16 h.

### 4.5. Swarming Motility Assay

Swarming motility assays were performed on 0.5% (*w/v*) agar M9 plates as described previously [51] with some modifications. Swarming medium was comprised of 62 mM potassium phosphate buffer (pH 7), 7 mM (NH_4_)_2_SO_4_, 2 mM MgSO_4_, 10 µM FeSO_4_, 0.4% (*w/v*) glucose, and 0.5% (*w/v*) casamino acids (Difco), solidified with 0.5% (*w/v*) Bacto-agar (Difco) and supplemented with either aspartame (1.36 mM), sucralose (25.2 mM), or saccharine (2.72 mM). After a brief solidification of the swarm plates, 1 μL *P. aeruginosa* PAO1 strain inoculum was spotted on the agar surface (center) enabling visualization of motility across the soft agar. *P. aeruginosa* PAO1 was grown previously overnight in M9 medium, then diluted 1:10 with fresh M9 medium and incubated at 37 °C with agitation until it reached mid-logarithmic phase (OD_600_ = 0.4–0.6). Swarming plates were incubated face up at 37 °C for 16–18 h. To quantify the degree of swarming, an image of the swarming plate was captured with a digital camera, and percent coverage of the plate was measured by ImageJ software version 1.8.0 (National Institutes of Health, Bethesda, MD, USA) [22].

### 4.6. QS Competition Assay Using Chromobacterium Violaceum CV026

The QS-inhibitory activity of artificial sweeteners was quantified by a flask incubation assay using activated CV026. Violacein was extracted by the method previously described [52] with slight modification. CV026 was grown for 24 h at 30 °C and back inoculated into a fresh LB medium to obtain an OD_600_ of 0.08, after which 32 μM *N*-hexanoyl-dl-homoserine lactone (HHL) was added along with the artificial sweeteners to the culture. The flasks were incubated for 20 h at 30 °C with constant agitation at 100 rpm. To quantify violacein, 1 mL of each reaction culture was centrifuged at 13,840× *g* for 10 min. The cell-free culture supernatants were removed and 1 mL DMSO was added to the pellet. The tubes were vortexed vigorously followed by centrifugation at 13,840× *g* for 5 min before the absorbance (at 585 nm) of the purple pigment was determined.

### 4.7. LasR-LBD Protein Expression, Purification, and Determination

Protein expression was performed in the *E. coli* BL21 strain transformed with a pETM-11 vector encoding a shortened His_6_-tagged LasR construct (LasR-LBD) spanning residues Met1 to Lys173 using 0.4 mM IPTG at 20 °C overnight in the presence of 7.5 μM 3-oxo-C12-HSL or/and the different concentrations of the artificial sweeteners. The cell pellets were obtained at 6000 rpm and resuspended in lysis buffer (50 mM Na_2_HPO_4_, 300 mM NaCl, 10 mM imidazole, pH 8) containing 1 mg mL^–1^ lysozyme and 1 mM PMSF. The resuspended cells were lysed by sonication for 40 s (4-s intervals) with 3-s pulses off at 30% amplitude. The lysate was centrifuged at 13,000× *g* rpm and the soluble fraction was obtained for purification by nickel-affinity chromatography and monitored by SDS–PAGE and Western blotting.

### 4.8. Microscale Thermophoresis (MST)

The full-length LasR protein was expressed and purified in the absence of AHL according to the protocol described by Krell and colleagues [39]. After the purification, the protein was monitored by SDS–PAGE, and fluorescently labeled using Monolith NanoTemper (NT^TM^) Protein Labeling Kit GREEN. The labeled protein (at a concentration of 0.5 µM) was titrated against the concentration gradient of 1 mM 3-oxo-C12-HSL and artificial sweeteners (aspartame, saccharin, and sucralose). The molecular interactions between the protein and the ligands (3-oxo-C12-HSL and artificial sweeteners) were measured in standard MST glass capillary tubes in a Monolith NT 115 instrument (NanoTemper Technologies GmbH, Munich, Germany) at a LED power of 20% [38]. The optimized buffer used was 50 mM sodium phosphate buffer, at pH 7. Monolith NT.115 analysis software (Nano Temper Technologies, Muüchen, Germany) was used to process the data and the *K_d_* values obtained were presented alongside the estimated standard error.

### 4.9. Molecular Docking

The atomic coordinates of the biological assembly (1) of the *P. aeruginosa* LasR-LBD in complex with its autoinducer 3-oxo-C12-HSL [23] (PDB entry: 2UV0; resolution: 1.80 Å; *R*-value, free: 0.254; *R*-value, work: 0.209) were downloaded from the RCSB Protein Data Bank [53] (available at https://www.rcsb.org/ [accessed 09 July 2020]) and provided as input to the Dock Prep utility of UCSF Chimera, version 1.11.2 [54] for receptor preparation purposes. The receptor was prepared by (i) removing solvent; (ii) mutating selenomethionine residues into methionine residues; (iii) adding hydrogen atoms; and (iv) removing chain E and 3-oxo-C12-HSL. The canonical or the isomeric (if applicable) SMILES string of each artificial sweetener was retrieved from the PubChem Substance and Compound databases [55] (available at https://pubchem.ncbi.nlm.nih.gov/ [accessed 22 July 2020]) and used as input to the Chemicalize online platform (ChemAxon Ltd., Budapest, Hungary) to identify the major macrospecies at physiological pH. After the SMILES strings were modified accordingly, they were submitted to the web-based SMILES translation service of the NCI/CADD group (available at https://cactus.nci.nih.gov/translate/ [accessed 3 September 2020]) to translate them into SDF files with three-dimensional coordinates. The binding modes and affinities of the artificial sweeteners of interest were predicted using an AutoDock Vina-based blind docking approach adopted by the CB-Dock web server, that automatically detect potential binding sites, estimating their centers and sizes, and customizing the grid box size based on query ligands [56] (available at http://cao.labshare.cn/cb-dock/ [accessed 28 September 2020]). The docking solutions were prioritized based on the AutoDock Vina scores, and favorable non-covalent interactions between LasR and the selected artificial sweeteners were calculated using Discovery Studio Visualizer, version 16.1.0 (Dassault Systèmes BIOVIA Corp., San Diego, CA, USA).

### 4.10. Statistical Analysis

GraphPad Prism Software version 6.00 for Windows (La Jolla, CA, USA) was used to perform all statistical analyses. Student’s *t*-test was used to compare all tests with the controls and calculations of the *p*-values. On the graphs, each data point represents the average of three different experimental readings to ensure the repeatability/reproducibility of the results. Values are expressed as mean ± standard deviation (SD).

## Data Availability

The data that support the findings of this study are available upon reasonable request from the authors.

## Supplementary Materials

The following are available online at https://www.mdpi.com/article/10.3390/ijms22189863/s1, Figure S1: Anti-QS activity of artificial sweeteners relative to the controls, corresponding to data in Figure 1, Figure S2: Effect of artificial sweeteners on K802NR growth, Figure S3: Response of PAO-JP2 (pKD-*rhlA*) reporter strain to different concentrations of artificial sweeteners, Figure S4: Inhibitory activity of saccharin against *Chromobacterium violaceum* CV026, Table S1: Profile of SS products.
