# Supplementary material for: Inhibitory Effects of Artificial Sweeteners on Bacterial Quorum Sensing
Source: Int J Mol Sci. 2021 Sep 13;22(18):9863. doi: 10.3390/ijms22189863 (PMC8472786; doi:10.3390/ijms22189863)
Supplement: Supplementary file 1 [file ijms-22-09863-s001.zip › ijms-1373797-supplementary.pdf]

International Journal of *Molecular Sciences*

MDPI

SUPPLEMENTARY MATERIALS FOR

Inhibitory Effects of Artificial Sweeteners on Bacterial Quorum Sensing

Victor Markus [1,2], Orr Share [2], Marilou Shagan [2], Barak Halpern [2], Tal Bar [2], Esti Kramarsky-Winter [2], Kerem Teralı [3], Nazmi Özer [4], Robert S. Marks [2,5], Ariel Kushmaro [2,5,6,*] and Karina Golberg [2,*]

[1] Department of Medical Biochemistry, Faculty of Medicine, Near East University, Nicosia 99138, Cyprus; victor.markus@neu.edu.tr
[2] Avram and Stella Goldstein-Goren Department of Biotechnology Engineering, Ben-Gurion University of the Negev, Be'er Sheva 84105, Israel; orrs@post.bgu.ac.il (O.S.); marilous@bgu.ac.il (M.S.); halpbar@post.bgu.ac.il (B.H.); bta@post.bgu.ac.il (T.B.); esti.winter@gmail.com (E.K.-W.); rsmarks@bgu.ac.il (R.S.M.)
[3] Department of Medical Biochemistry, Faculty of Medicine, Girne American University, Kyrenia 99428, Cyprus; keremterali@gau.edu.tr
[4] Department of Biochemistry, Faculty of Pharmacy, Girne American University, Kyrenia 99428, Cyprus; nazmiozer@gau.edu.tr
[5] The Ilse Katz Center for Nanoscale Science and Technology, Ben-Gurion University of the Negev, Be'er Sheva 84105, Israel
[6] School of Sustainability and Climate Change, Ben-Gurion University of the Negev, Beer-Sheva 84105, Israel
* Correspondence: arielkus@bgu.ac.il (A.K.); karingo@post.bgu.ac.il (K.G.); Tel.: +972-747795291 (A.K.); +972-74-7795293 (K.G.)

Table S1. Profile of SS products

SS product	Ingredients	Artificial sweetener present	Recommended Amount (1 oz = 30 mL)
SS1	Cellulose, Creatine Hydrochloride, Dicalcium phosphate, Natural Mint Flavor, Enteric Coating (Sodium, Cellulose, Alginate, Stearic Acid, Oleic Acid, and Medium Chain Triglycerides), Titanium Dioxide and Sucralose.	Sucralose	2 tablets (5 g)
SS2	Potassium, Calcium, Sodium, Dietary Fibers, Protein, Cholesterol, Saturated Fat, Trans Fat, Sugars, Cocoa (Processed with Alkali), Acesulfame Potassium, Sucralose, Enzyme Blend (Aminogen®, Lactase), Natural and Artificial Flavors, Lecithin, Salt, Whey Protein Blend (Whey Protein Concentrate, Whey Protein Isolate, Whey Protein Hydrolysate), Xanthan Gum	Ace-K and Sucralose	1 (31 g) to 2 (62 g) scoops in 6–8 oz
SS3	Calcium, Iron, Sodium, Dietary Fiber, Cholesterol, Protein, Sugar, Saturated Fat, Alkalized Cocoa Powder, Sucralose, Acesulfame-Potassium, Calcium Carbonate, Gum Blend (Xanthan Gum, Cellulose Gum, Carrageenan), Salt, Natural and Artificial Flavors, Soy Lecithin, Sunflower-based Creamer (Corn syrup solids, Sunflower oil, Sodium Caseinate, Tocopherols, Mono-Diglycerides, Dipotassium Phosphate), Whey Protein Isolate, whey Protein Concentrate, Whey Peptides, Tricalcium Phosphate.	Ace-K and Sucralose	1 (34 g) scoop in 6 oz skim milk or water
SS4	Potassium, Sodium, Calcium, Iron, Dietary Fiber, Cholesterol, Saturated Fat, Trans Fat, Protein, Sugar, Vitamin C, Vitamin A, Amino Matrix (L-Taurine, L-Glycine, BCAAs (Leucine, Valine, Iso-Leucine), L-Glutamine), Acesulfame-Potassium, Sucralose, Flax Seed Oil, Lactase, Glucose Polymers, Natural and Artificial Flavors, Suspension Matrix (Xanthan Gum, Guar Gum, Cellulose Gum), Sea Salt, Whey Protein Isolate, Whey Protein Concentrate, Whey Protein Hydrolysate	Ace-K and Sucralose	1 (32.4 g) to 2 (64.8 g) scoops in 8–12 oz
SS5	Calcium, Potassium, Sodium, Iron, Dietary Fiber, Cholesterol, Multi-level Amino Acid Growth Matrix, Saturated Fat, Protein, Trans Fat, Methionine, Tyrosine, Serine, Threonine, Arginine, Alanine, Glycine, Aspartic Acid, Lysine, Phenylalanine, Cystine, Tryptophan, Proline, Histidine, BCAAs (L-Leucine, L-Glutamine, L-isoleucine, L-valine), Egg Albumen, Digestive Enzyme Blend, Micellar Casein, Lactase, Partially hydrolyzed	Ace-K and Sucralose	1 (34.9 g) to 2 (69.8 g) scoops in 8–12 oz

	Whey Concentrate, Whey Protein Isolate, Whey Protein Concentrate, Protease.

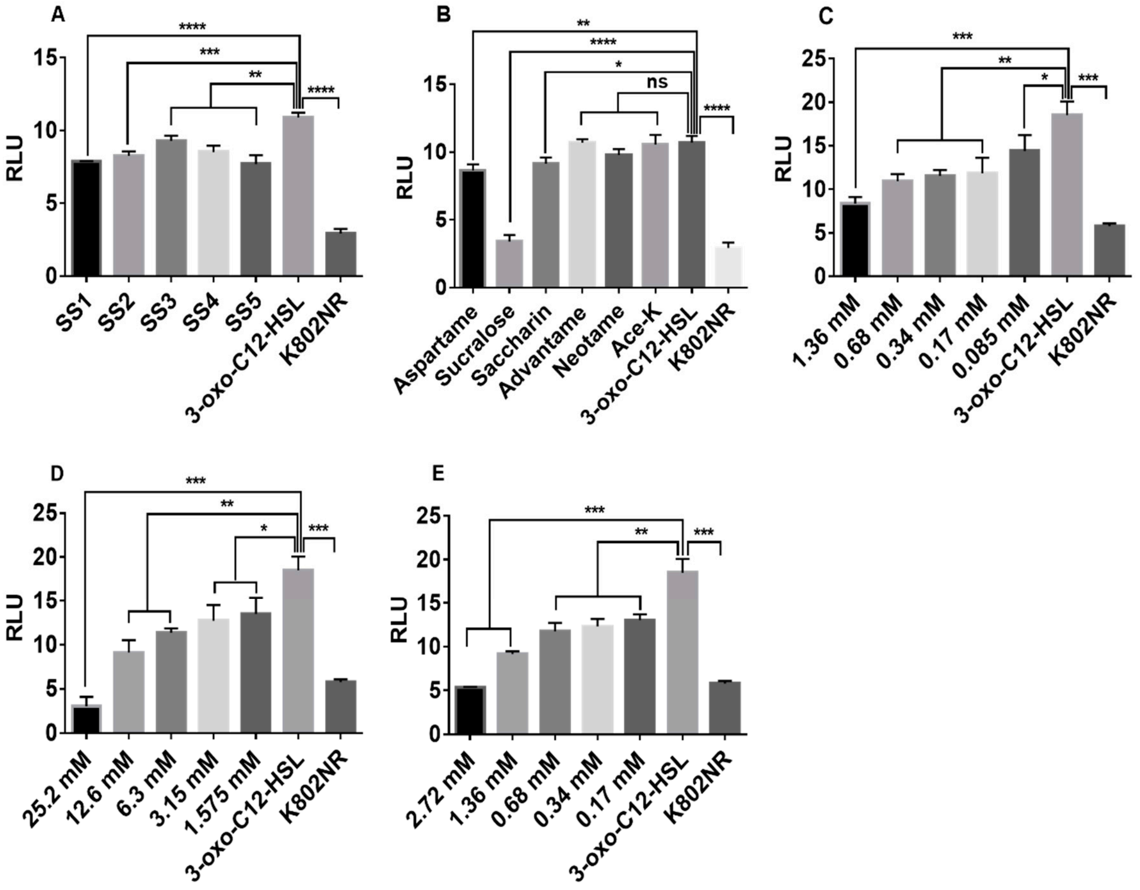

Figure S1. Anti-QS activity of artificial sweeteners relative to the controls, corresponding to data in Figure 1. **(A)** Effect of SS products on K802NR, corresponding to data in Figure 1A. SS1 (0.2 mg mL^{-1}) contains sucralose, SS2 (0.4 mg mL^{-1}) contains Ace-K and sucralose, SS3 (0.2 mg mL^{-1}) contains Ace-K and sucralose, SS4 (0.3 mg mL^{-1}) contains Ace-K and sucralose, and SS5 (0.3 mg mL^{-1}) contains Ace-K and sucralose. The amounts of artificial sweeteners in the products are undisclosed. **(B)** Screening of six pure FDA approved artificial sweeteners, corresponding to data in Figure 1B. Aspartame (1.36 mM), sucralose (25.2 mM), saccharin (2.72 mM), advantame (0.42 mM), neotame (0.53 mM) and Ace-K (4.97 mM). **(C)** The response of K802NR to different concentrations of aspartame, corresponding to data in Figure 1C. **(D)** The response of K802NR to different concentrations of sucralose, corresponding to data in Figure 1D. **(E)** The response of K802NR to different concentrations of saccharin, corresponding to data in Figure 1E. All concentrations of artificial sweeteners presented are final concentrations. The final concentration of 3-oxo-C12-HSL used was 5×10^{-10} M. The luminescence of K802NR was expressed as RLU. * $p < 0.05$, ** $p < 0.01$, *** $p < 0.001$, **** $p < 0.0001$, and *ns* not significant. Values are mean ± SD, $n = 3$ (three different experimental readings).

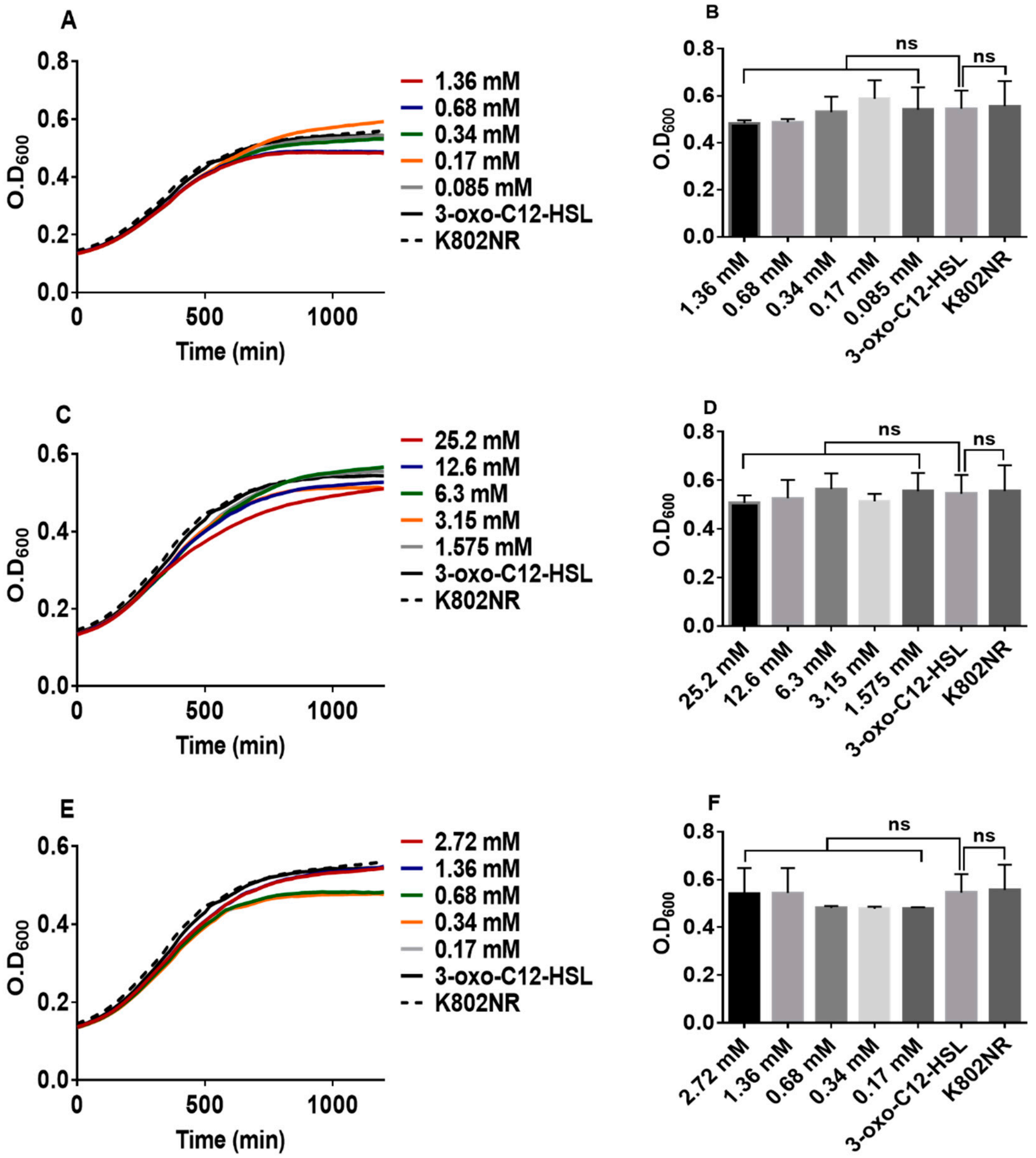

Figure S2. Effect of artificial sweeteners on K802NR growth. **(A)** aspartame; (**B**) aspartame relative to the control, consistent with the data in panel A; **(C)** sucralose; (**D**) sucralose relative to the control, consistent with the data presented in panel C; **(E)** saccharin; **(F)** saccharin relative to the control, consistent with the data presented in panel E. All concentrations of artificial sweeteners presented are final concentrations. The final concentration of 3-oxo-C12-HSL used was 5×10^{-10} M. Values are mean ± SD, *n* = 3 (three different experimental readings), ns not significant.

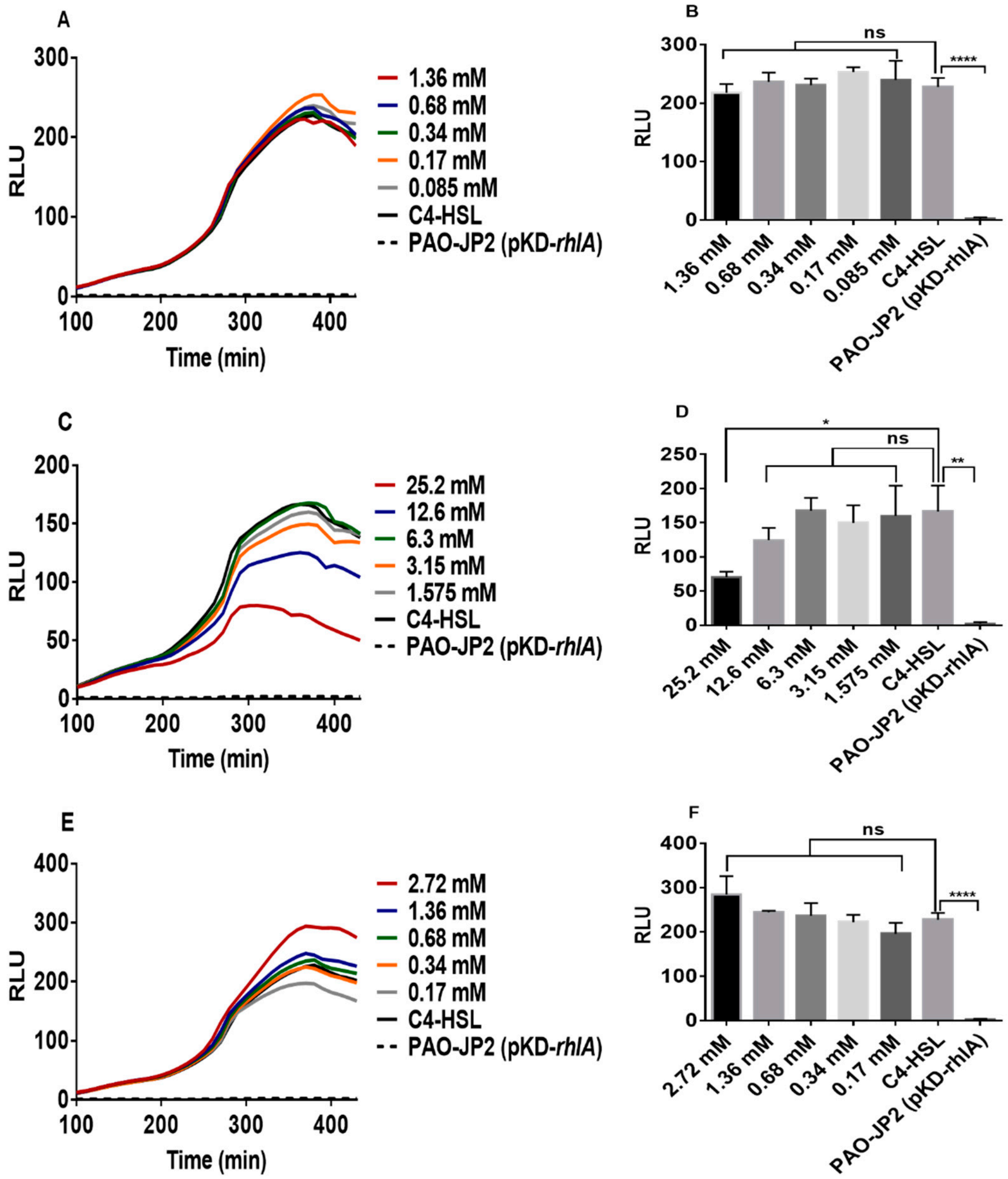

Figure S3. Response of PAO-JP2 (pKD-*rhlA*) reporter strain to different concentrations of artificial sweeteners: **(A)** aspartame; **(B)** aspartame relative to control, correlating to data in panel A; **(C)** sucralose; **(D)** sucralose relative to control, correlating to data in panel C; **(E)** saccharin; **(F)** saccharin relative to control, correlating to data in panel E. All concentrations of artificial sweeteners presented are final concentrations. The final concentration of C4-HSL used was 10 µM. The luminescence of K802NR was expressed as RLU. * $p < 0.05$, and *ns* not significant. Values are mean ± SD, $n = 3$ (three different experimental readings).

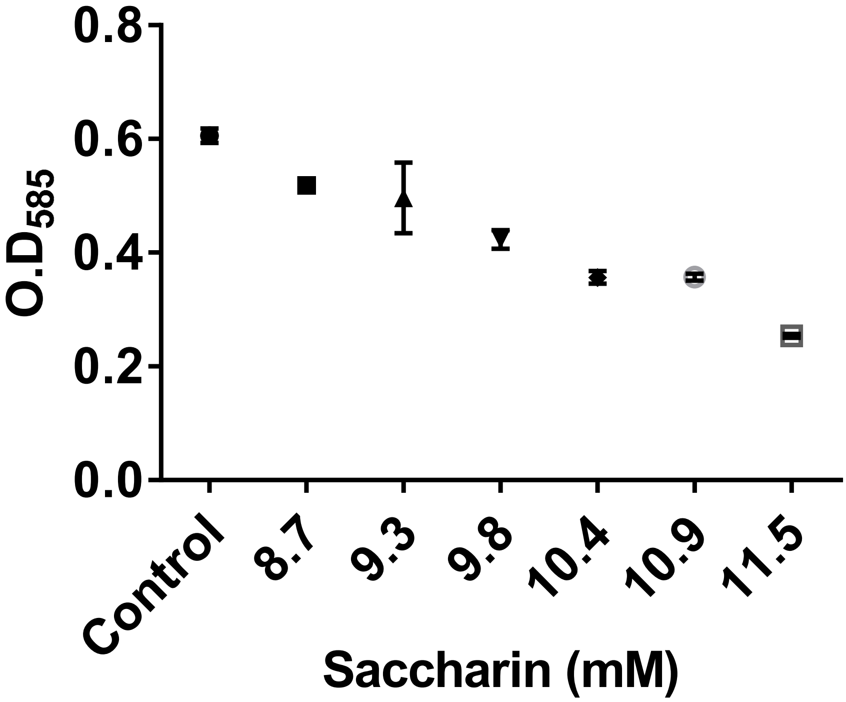

Figure S4: Inhibitory activity of saccharin against *Chromobacterium violaceum* CV026
